# Two new cestode species of *Tetragonocephalum* Shipley & Hornell, 1905 (Lecanicephalidea, Tetragonocephalidae) from *Himantura randalli* Last, Manjaji-Matsumoto & Moore (Myliobatiformes, Dasyatidae) from the Gulf of Oman

**DOI:** 10.3897/zookeys.623.9724

**Published:** 2016-10-11

**Authors:** Atabak Roohi Aminjan, Masoumeh Malek

**Affiliations:** 1 School of Biology and Center of Excellence in Phylogeny of Living Organisms, College of Science, University of Tehran, Enghelab Ave., Tehran, Iran

**Keywords:** Tetragonocephalum sabae sp. n., Tetragonocephalum salarii sp. n., Himantura randalli, Gulf of Oman

## Abstract

The original description of the genus *Tetragonocephalum* was published more than one hundred years ago but its taxonomic status was clarified only recently. To date, approximately 30 nominal species of this genus have been described, mostly from the northern Indian Ocean, but nearly half of them are invalid and only 14 species are recognized as valid. In the present study two new species of *Tetragonocephalum* are described from the spiral intestine of *Himantura randalli* from off Jod, on the northern coast of the Gulf of Oman. *Tetragonocephalum sabae* **sp. n.** is distinguishable from the valid species of *Tetragonocephalum* based on number of proglottids (43−53), number of testes (42−50), and size of scolex (401−453×328−455), acetabula (87−109×72−116), mature proglottids (802−1,333×226−336), cirrus sac (92−160×103−154), and eggs (16−19×11−13). *Tetragonocephalum salarii* **sp. n.** can be distinguished from *Tetragonocephalum sabae* **sp. n.** and all other valid species of *Tetragonocephalum* based on number of proglottids (77−86). Furthermore, it differs from its congeners based on a combination of some characteristics, including the number of mature (3−7) and gravid (18−20) proglottids, the number of testes (30−38), and the size of acetabula (84−111×80−96), mature proglottids (497−833×334−403), gravid proglottids (1,036−1,482×440−575), testes (20−34×31−50), ovary (123−215×210−278), and eggs (24−45×13−21).

## Introduction

Shipley and Hornell (1905) erected *Tetragonocephalum* and described two new species, the type species *Tetragonocephalum trygonis* Shipley & Hornell, 1905 from *Himantura walga* (Müller & Henle) (as *Trygon walga*) and *Tetragonocephalum aetiobatidis* Shipley & Hornell, 1905 from *Aetobatus ocellatus* (Kuhl) (as *Aetiobatis* [sic] *narinari*), collected from fishes taken from the Gulf of Manaar off the coast of Ceylon (now Sri Lanka). Later, a number of species of this genus were described from several different localities around the world, mostly from the Northern Indian Ocean (Jensen 2005).

Jensen (2005), who revised the order Lecanicephalidea, resolved the taxonomic status of *Tetragonocephalum*, which she considered to include 14 valid species (13 listed and a type species), i.e., *Tetragonocephalum alii* Deshmukh & Shinde, 1979, *Tetragonocephalum aurangabadensis* Shinde & Jadhav, 1990, *Tetragonocephalum bhagawatii* Shinde, Mohekar & Jadhav, 1985, *Tetragonocephalum madhulatae* (Andhare & Shinde, 1994) Jensen, 2005, *Tetragonocephalum madrasensis* (Andhare & Shinde, 1994) Jensen, 2005, *Tetragonocephalum passeyi* Jensen, 2005, *Tetragonocephalum raoi* Deshmukh & Shinde, 1979, *Tetragonocephalum ratnagiriensis* Shinde & Jadhav, 1990, *Tetragonocephalum sephenis* Deshmukh & Shinde, 1979, *Tetragonocephalum shipleyi* Shinde, Mohekar & Jadhav, 1985, *Tetragonocephalum simile* (Pintner, 1928) Ivanov & Campbell, 2000, *Tetragonocephalum trygonis*, *Tetragonocephalum uarnak* (Shipley & Hornell, 1906) Pintner, 1928, and *Tetragonocephalum yamagutii* Muralidhar, 1988; in addition, she recognized three *species inquirendae*, and five species as *nomina nuda*. Since 2006, some new species of *Tetragonocephalum* have been proposed, i.e., *Tetragonocephalum govindi* Khamkar & Shinde, 2012 (Khamkar and Shinde 2012); *Tetragonocephalum panjiensis* Khamkar, 2011 (Khamkar 2011); *Tetragonocephalum pulensis* Kankale, 2014 (Kankale 2014); *Tetragonocephalum ratnagiriensis* Khamkar, 2012 (Khamkar 2012); *Tetragonocephalum sepheni* Lanka, Hippargi & Patil, 2013 (Lanka et al. 2013). These species do not follow the rules of ICZN, and especially violate Article 16, hence they are unavailable (ICZN 1999).

The only study on *Tetragonocephalum* from the Gulf of Oman was conducted by Golestaninasab et al. (2014) who showed that the genus *Tetragonocephalum* can act as a heavy metal bioindicator in the marine environment.

The present article is the first taxonomic study of the genus *Tetragonocephalum* from the Gulf of Oman. We describe two new species of this genus collected from the spiral intestine of the Arabian banded whipray, *Himantura randalli* Last, Manjaji-Matsumoto & Moore, 2012.

## Materials and methods

A total of 36 specimens of *Himantura randalli* was collected from northern waters of the Gulf of Oman, 29 individuals in May 2011 and seven in October 2012. They were caught by local fishermen off Jod, Zarabad, Iran (25°26'58"N, 59°30'29"E). Each specimen was given a unique collection number for author reference. All host individuals were photographed and morphometric and morphological characteristics were recorded to facilitate species identification. Species identity was confirmed using Naylor et al. (2012), Last et al. (2012), and Henderson et al. (2015).

Host specimens were dissected along the mid-ventral line; spiral intestines were removed and opened by a longitudinal incision. Subsequently, spiral intestines were fixed in 10% seawater buffered formalin, shaken vigorously, and held for approximately seven days. The samples were then transferred to the Zoology Laboratory, School of Biology, University of Tehran for detailed examination of parasitic infection.

Spiral intestines and intestinal contents were examined under a stereomicroscope. Tapeworms were carefully removed from the spiral intestine and washed in distilled water for about one hour before being preserved in 70% ethanol. Parasite specimens were prepared as whole mounts for light microscopy observation according to Koch et al. (2012).

Whole mounts were studied using a Leica DM500 light microscope. Images of *Tetragonocephalum* specimens were taken using a Leica ICC50 HD color digital camera mounted on the Leica DM500 light microscope (Buffalo Grove, Illinois, United States) and measurements were taken using the image analysis software Leica Application Suite (LAS EZ v.3.0.0) (Leica 2013). Measurements were analyzed in IBM® SPSS® Statistics Package v.22 (IBMCorp. 2013). All measurements of the reproductive organs were taken from mature proglottids. Measurements are given in micrometers (µm) unless otherwise indicated; they are given as the range followed in parentheses by the mean, number of worms examined, and the total number of measurements if more than one measurement was taken per worm. Illustrations were prepared with Adobe® Illustrator® CC (Adobe Incorp. 2013) based on the drafts sketched under a Reichert Biovar microscope with the aid of a drawing tube.

Some scoleces were prepared for ultrastructural studies using SEM following the protocol of Jensen (2005). The specimens were sputter coated with approximately 10 nm of gold/palladium, and examined with a field emission scanning election microscope (HIT4160102, Hitachi, Tokyo, Japan) at the School of Electrical and Computer Engineering (ECE), University of Tehran. Microthrix terminology follows Chervy (2009).

Type and voucher specimens are deposited at The Zoological Museum, University of Tehran, Tehran, Iran (ZUTC), and The Natural History Museum, London, England (BMNH).

## Results

### Tetragonocephalum sabae sp. n.

Taxon classificationAnimaliaMyliobatiformesDasyatidae

http://zoobank.org/5FA0CE20-885D-4433-9B69-1C830667FD86

#### Type-materials.

**Holotype**: Slide. Original label: “*Tetragonocephalum sabae*; Roohi Aminjan & Malek; Holotype; Canada balsam; ID: A. Roohi Aminjan & M. Malek; Scolex, proglottid & worm drawn; ZUTC Platy. 1500; (AR-1009u); ex *Himantura randalli*; spiral intestine; Gulf of Oman, Iran; Coll. May 2011”; Zoological Museum, University of Tehran, Tehran (ZUTC). **Paratypes**: Two slides, ZUTC Platy. 1501 and ZUTC Platy. 1503, from the same host individual as the holotype. **Other materials**: Two scoleces for SEM, ZUTC Platy. 1502s and ZUTC Platy. 1504s (two stubs) and their whole-mounted vouchers, ZUTC Platy. 1502v and ZUTC Platy. 1504v (two slides), from the same host individual as the holotype.

#### Type locality.

Off Jod (25°26'58"N; 59°30'29"E), Zarabad, Iran, Gulf of Oman. Other localities: None.

#### Type host.

*Himantura randalli* Last, Manjaji-Matsumoto & Moore, 2012, the Arabian banded whipray (Myliobatiformes, Dasyatidae) [host no. MM1009]. Additional hosts: None. Site of infection: Spiral intestine. Prevalence: 2.78% (one of 36 individuals examined). Intensity: Five specimens.

#### Diagnosis.

*Tetragonocephalum sabae* sp. n. can be distinguished from all the other valid species of *Tetragonocephalum* by the number of proglottids and testes, the size of scolex, acetabula, mature proglottids, cirrus sac, and eggs.

#### Description

**Figure 1.**
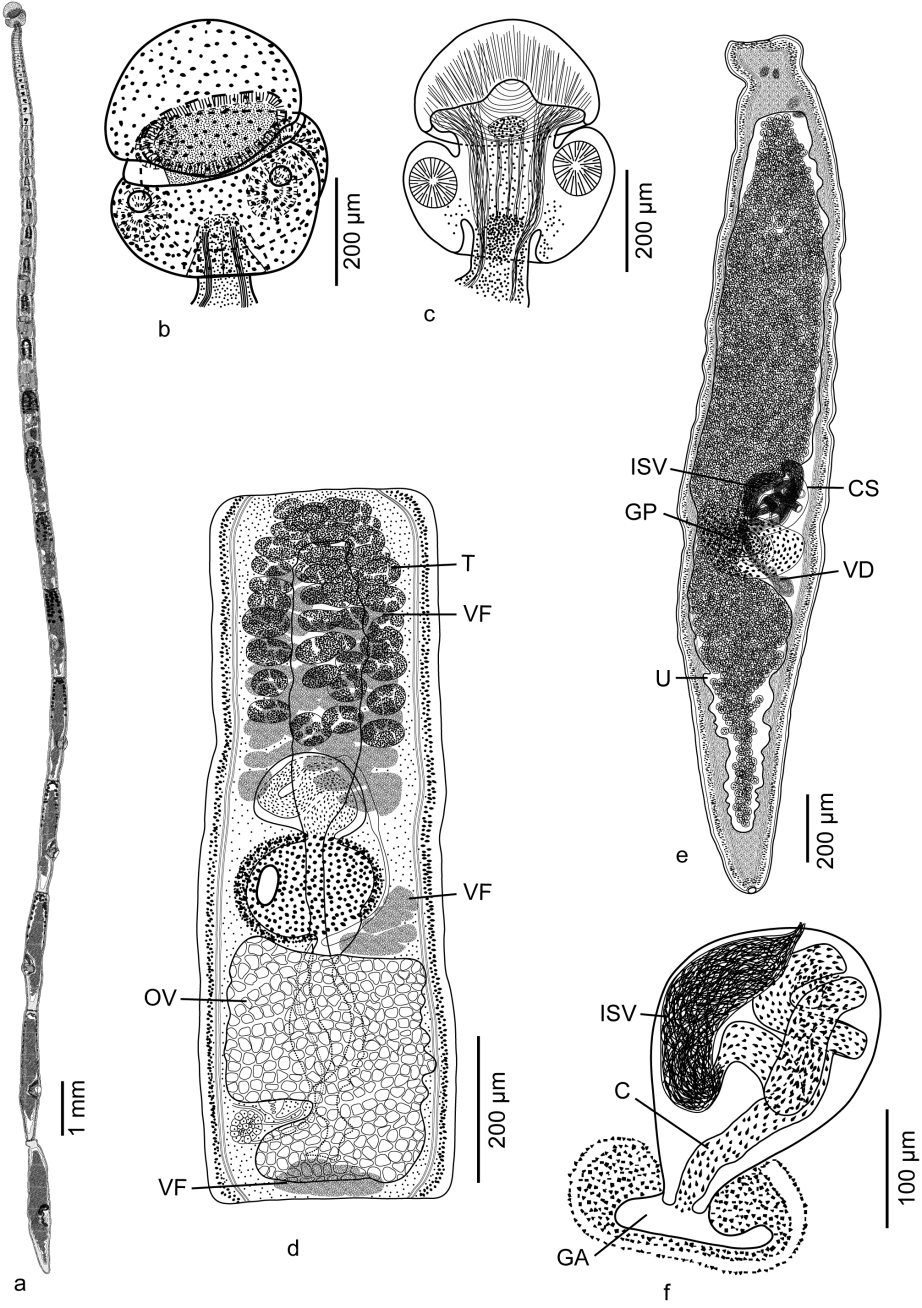
Line drawings of *Tetragonocephalum sabae* sp. n. from *Himantura randalli*. **a** Whole worm **b** Whole structure of scolex **c** Internal details of scolex **d** Mature proglottid **e** Gravid proglottid **f** Cirrus sac. Abbreviations: C, Cirrus; CS, Cirrus Sac; GA, Genital Atrium; GP, Genital Pore; ISV, Internal Seminal Vesicle; T, Testis; OV, Ovary; U, Uterus; VD, Vas Deferens; VF, vitelline follicle.

**(Figures 1 and 3a−d).** Based on three whole mounts of gravid specimens and two scoleces prepared for SEM and their vouchers (partially measured).

Worms 23.2−32.4 (27.5; 3) mm long, apolytic; maximum width 398−489 (425; 5) at posterior-most gravid proglottid; with 42−53 (48; 3) proglottids (Figure 1a). Scolex 401−453 (426; 5) long by 328−455 (383; 5) wide, consisting of scolex proper and apical organ. Scolex proper 197−267 (234; 5) long by 328−455 (383; 5) wide, bearing four acetabula. Acetabula sucker-like in form, 87−109 (94; 3; 12) long by 72−116 (98; 3; 12) wide. Apical modification of scolex proper cylindrical, bearing apical organ. Apical organ muscular, with glandular surface, 178−216 (198; 5) long by 321−352 (344; 5) wide, non-invaginable, non-retractable (Figures 1b−c and 3a).

Apical organ covered with tubercles suggestive of glandular surface (Figure 3b). Microtriches on apical modification of scolex proper and scolex proper not observed (Figure 3c). Strobila covered with capilliform filitriches (Figure 3d).

Cephalic peduncle absent. Proglottids acraspedote. Immature proglottids 32−41 (36; 3) in number, initially wider than long, gradually becoming longer than wide (5−8 [7; 3] of immature proglottids longer than wide); two posterior-most immature proglottids 583−857 (746; 5; 10) long by 245−314 (278; 5; 10) wide. Mature proglottids 2 (2; 5) in number; two posterior-most mature proglottids 802−1,333 (1,074; 5; 10) long by 226−336 (287; 5; 10) wide (Figure 1d). Gravid proglottids 8−10 (9; 4) in number; two posterior-most gravid proglottids 2,311−2,910 (2,522; 4; 8) long by 398−489 (425; 4; 8) wide (Figure 1e).

Testes oval to spherical, 42−50 (46; 5; 10) in number, 28−47 (40; 5; 30) long by 37−57 (47; 5; 30) wide, extending from anterior margin of proglottid to anterior margin of cirrus sac, in multiple irregular columns in dorso-ventral view, four layers deep in cross section. Vas deferens extending from level of anterior margin of ovary to cirrus sac, entering cirrus sac at distal end (Figure 1d). External seminal vesicle absent. Internal seminal vesicle present, visible in gravid proglottids. Cirrus sac oval or spherical in form, angled anteriorly, 92−160 (123; 5; 10) long by 103−154 (124; 5; 10) wide, containing coiled cirrus. Cirrus armed with spinitriches (Figure f). Ovary oblong in dorso-ventral view, incomplete ring-shaped in cross-section, 290−428 (357; 5; 10) long by 175−279 (217; 5; 10) wide, symmetrical. Mehlis’ gland posterior to ovarian bridge, 41−83 (60; 5; 10) long by 43−76 (59; 5; 10) wide. Vagina extending along median line from ootype to genital atrium, opening into genital atrium posterior to cirrus sac; vaginal sphincter absent. Genital pores lateral (sub-marginal in some segments, Figure 1d), irregularly alternating, 37−43% (40; 5; 10) of proglottid length from posterior end. Genital atrium expanded, conspicuous. Uterus bisaccate, extending along median line of proglottid from posterior margin of ovary to anterior margin of proglottid, constricted at level of genital atrium; uterine duct entering uterus at level of posterior margin of genital atrium. Vitellarium follicular; vitelline follicles medullary, 28−40 (33; 5; 30) long by 57−73 (64; 5; 30) wide, in three fields; anterior field anterior to genital atrium stopping short of anterior margin of proglottid; middle field between genital atrium and ovary; posterior field posterior to ovary (Figure 1d). Osmoregulatory ducts in two lateral pairs. Eggs single, lacking polar filaments, 16−19 (17; 4; 12) long by 11−13 (12; 4; 12) wide, adhering to one another in uterus; embryonated in older gravid proglottids.

#### Etymology.

This species is named in honor of the first author’s wife, Saba Saadati Safa, for her unconditional support and patience over the last five years.

#### Remarks.

The possession of a bisaccate uterus and other characteristics clearly place this new species in the genus *Tetragonocephalum*. With respect to the 14 valid species of *Tetragonocephalum*, *Tetragonocephalum sabae* sp. n. has a greater number of testes (42−50) than *Tetragonocephalum bhagawatii* (37−38), *Tetragonocephalum sephenis* (36−38), *Tetragonocephalum shipleyi* (12) and fewer than *Tetragonocephalum aurangabadensis* (105−110), *Tetragonocephalum madrasensis* (125−130), *Tetragonocephalum passeyi* (54−73), *Tetragonocephalum raoi* (50−55), and *Tetragonocephalum yamagutii* (54−56). It possesses more proglottids (42−53) than *Tetragonocephalum uarnak* (30−40) and fewer than *Tetragonocephalum alii* (55−60), *Tetragonocephalum simile* (75), and *Tetragonocephalum trygonis* (60). *Tetragonocephalum sabae* sp. n. differs from *Tetragonocephalum madhulatae* in the size of the mature proglottids (802−1,333×226−336 *vs* 1,359−1,455×295−334) and eggs (16−19×11−13 *vs* 52−57×38−43); and from *Tetragonocephalum ratnagiriensis* in the size of the scolex (401−453×328−455 *vs* 843×469−537), acetabula (87−109×72−116 *vs* 130×111), and cirrus sac (92−160×103−154 *vs* 213×86−188).

### Tetragonocephalum salarii sp. n.

Taxon classificationAnimaliaMyliobatiformesDasyatidae

http://zoobank.org/5053B3A5-A29C-4E04-B2C6-D3AE518C31ED

#### Type-materials.

**Holotype**: Slide. Original label: “*Tetragonocephalum salarii*; Roohi Aminjan & Malek; Holotype; Canada balsam; ID: A. Roohi Aminjan & M. Malek; Scolex, proglottid & worm drawn; ZUTC Platy. 1546; (AR-1245c); ex *Himantura randalli*; spiral intestine; Gulf of Oman, Iran; Coll. Oct 2012”; Zoological Museum, University of Tehran, Tehran (ZUTC). **Paratypes**: Two slides, ZUTC Platy. 1547 and ZUTC Platy. 1548, from the same host individual as the holotype. **Other materials**: One scolex for SEM, ZUTC Platy. 1549s (one stub) and its whole-mounted voucher, ZUTC Platy. 1549v (one slide), from the same host individual as the holotype.

#### Type locality.

Off Jod (25°26'58"N; 59°30'29"E), Zarabad, Iran, Gulf of Oman. Other localities: None.

#### Type host.

*Himantura randalli* Last, Manjaji-Matsumoto & Moore, 2012, the Arabian banded whipray (Myliobatiformes, Dasyatidae) [host no. MM1245]. Additional hosts: none. Site of infection: spiral intestine. Prevalence: 2.78% (one of 36 individuals examined). Intensity: Four specimens.

#### Diagnosis.

*Tetragonocephalum salarii* sp. n. can be distinguished from *Tetragonocephalum sabae* sp. n. and all other valid species of *Tetragonocephalum* by the number of mature and gravid proglottids, the number of testes; and the size of acetabula, mature proglottids, gravid proglottids, testes, ovary, and eggs.

#### Description

**Figure 2.**
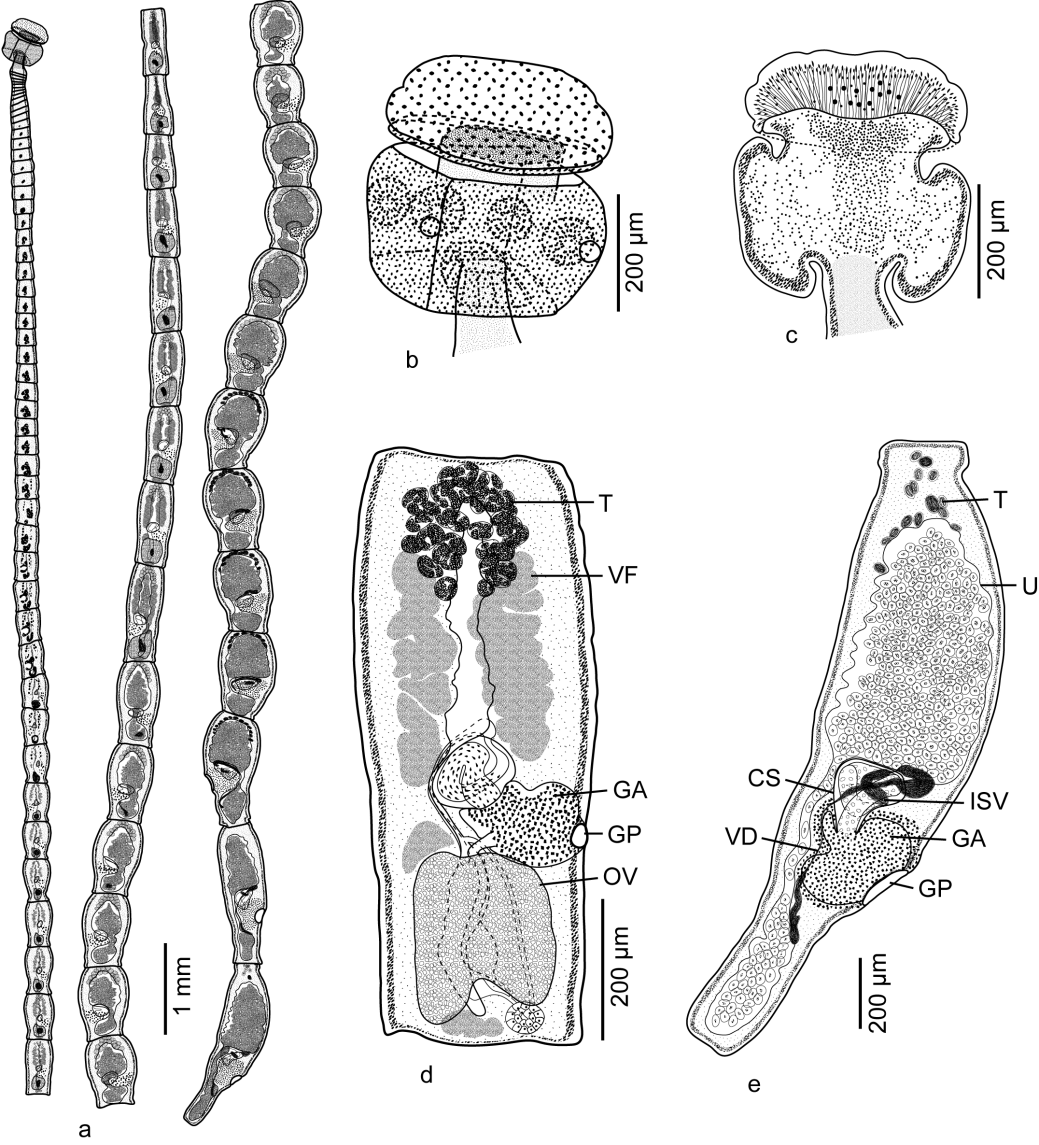
Line drawings of *Tetragonocephalum salarii* sp. n. from *Himantura randalli*. **a** Whole worm **b** Whole structure of scolex **c** Internal details of scolex **d** Mature proglottid **e** Gravid proglottid. Abbreviations: CS, Cirrus Sac; GA, Genital Atrium; GP, Genital Pore; ISV, Internal Seminal Vesicle; T, Testis; OV, Ovary; U, Uterus; VD, Vas Deferens; VF, vitelline follicle.

**Figure 3.**
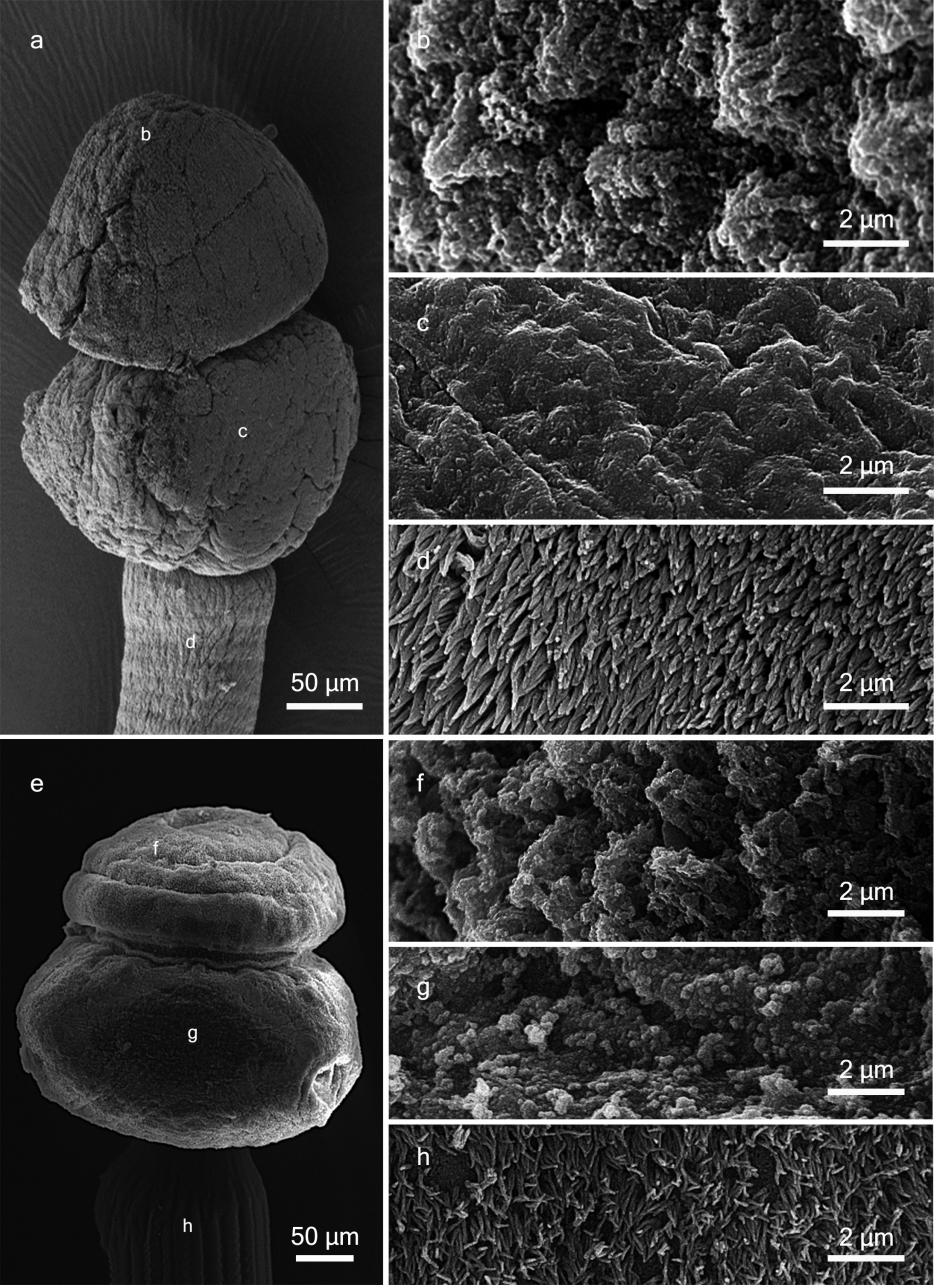
SEM micrographs of *Tetragonocephalum sabae* sp. n. (**a−d**) and *Tetragonocephalum salarii* sp. n. (**e−h**) from *Himantura randalli*. **a, e** Scolex **b, f** Tubercles on apical organ surface **c, g** Surface of scolex proper **d, h** Capilliform filitriches on strobila.

**(Figures 2 and 3e−h).** Based on three whole mounts of gravid specimens and one scolex prepared for SEM and its voucher (partially measured).

Worms 23.5−35.9 (27.8; 3) mm long, apolytic; maximum width 440−575 (517; 3) at posterior-most gravid proglottid; with 76−86 (80; 3) proglottids (Figure 2a). Scolex 453−501 (472; 3) long by 406−480 (441; 3) wide, consisting of scolex proper and apical organ. Scolex proper 258−300 (276; 3) long by 390−457 (426; 3) wide, bearing four acetabula. Acetabula sucker-like in form, 84−111 (96; 3; 12) long by 80−96 (90; 3; 12) wide. Apical modification of scolex proper cylindrical, bearing apical organ. Apical organ muscular, with glandular surface, 178−233 (201; 3) long by 406−480 (441; 3) wide, non-invaginable, non-retractable (Figures 2b−c and 3e).

Apical organ covered with tubercles suggesting glandular surface (Figure 3f). Microtriches not observed on apical modification of scolex or on scolex proper (Figure 3g). Strobila covered with capilliform filitriches (Figure 3h).

Cephalic peduncle absent. Proglottids acraspedote. Immature proglottids 55−62 (58; 3) in number, initially wider than long, gradually becoming longer than wide (7−21 [14; 3] immature proglottids longer than wide); two posterior-most immature proglottids 464−696 (523; 4; 8) long by 295−393 (346; 4; 8) wide. Mature proglottids 3−7 (5; 4) in number; two posterior-most mature proglottids 497−833 (672; 4; 8) long by 334−403 (365; 4; 8) wide (Figure 2d). Gravid proglottids 18−20 (19; 3) in number; two posterior-most gravid proglottids 1,036−1,482 (1,239; 3; 6) long by 440−575 (517; 3; 6) wide (Figure 2e).

Testes oval, 30−38 (34; 4; 8) in number, 20−34 (26; 4; 24) long by 31−50 (40; 4; 24) wide, restricted to anterior quarter of proglottid, in multiple irregular columns in dorso-ventral view, four layers deep in cross section. Vas deferens extending from level of anterior margin of ovary to cirrus sac, entering cirrus sac at distal end (Figure 2d). External seminal vesicle absent. Internal seminal vesicle present. Cirrus sac oval in form, angled anteriorly, 53−88 (68; 4; 8) long by 120−160 (141; 4; 8) wide, containing coiled cirrus (Figures 2d−e). Cirrus armed with spinitriches. Ovary oblong to quadrate in dorso-ventral view, incomplete ring-shaped in cross-section, 123−215 (182; 4; 8) long by 210−278 (240; 4; 8) wide, symmetrical. Mehlis’ gland posterior to ovary, 39−59 (48; 4; 8) long by 48−62 (54; 4; 8) wide. Vagina extending along median line from ootype to genital atrium, opening into genital atrium posterior to cirrus sac; vaginal sphincter absent. Genital pores lateral, irregularly alternating, 30−36% (33; 4; 8) of proglottid length from posterior end. Genital atrium massive, conspicuous. Uterus bisaccate, extending along median line of proglottid from posterior margin of ovary to anterior margin of proglottid, constricted at level of genital atrium; uterine duct entering uterus at level of posterior margin of genital atrium. Vitellarium follicular; vitelline follicles medullary, 38−48 (44; 4; 24) long by 57−69 (65; 4; 24) wide, in three fields; anterior field anterior to genital atrium stopping short of anterior margin of proglottid; middle field generally between genital atrium and ovary; posterior field posterior to ovary (Figure 2d). Osmoregulatory ducts in two lateral pairs. Eggs single, lacking polar filaments, 24−45 (34; 3; 9) long by 13−21 (17; 3; 9) wide, adhering to one another in uterus; embryonated of older gravid proglottids.

#### Etymology.

This species is named in honor of Mr. Naser Salari in gratitude for his assistance with the collection of host specimens.

#### Remarks.

*Tetragonocephalum salarii* sp. n. possesses the characteristics of the genus and can be distinguished from *Tetragonocephalum sabae* sp. n. and all 14 valid congeners based on the following characteristics. *Tetragonocephalum salarii* sp. n. differs from *Tetragonocephalum sabae* sp. n. in the total number of proglottids (77−86 *vs* 42−53), mature proglottids (3−7 *vs* 2), gravid proglottids (18−20 *vs* 8−10), and testes (30−38 *vs* 42−50); and in the size of mature proglottids (497−833×334−403 *vs* 802−1,333×226−336), gravid proglottids (1,036−1,482×440−575 *vs* 2,311−2,910×398−489), and eggs (24−45×13−21 *vs* 16−19×11−13). It has a greater number of testes (30−38) than *Tetragonocephalum shipleyi* (12) and fewer than *Tetragonocephalum alii* (40−45), *Tetragonocephalum aurangabadensis* (105−110), *Tetragonocephalum madhulatae* (45), *Tetragonocephalum madrasensis* (125−130), *Tetragonocephalum passeyi* (54−73), *Tetragonocephalum raoi* (50−55), *Tetragonocephalum ratnagiriensis* (40−44), and *Tetragonocephalum yamagutii* (54−56). This new species has more proglottids (77−86) than *Tetragonocephalum sephenis* (20−25), *Tetragonocephalum simile* (75), *Tetragonocephalum trygonis* (60), and *Tetragonocephalum uarnak* (30−40). *Tetragonocephalum salarii* sp. n. differs from *Tetragonocephalum bhagawatii* in the size of the acetabula (84−111×80−96 *vs* 56 dia.), testes (20−34×31−50 *vs* 18 dia.), and gravid proglottids (1,036−1,482×440−575 *vs* 860−920×300−360).

## Discussion

The genus *Tetragonocephalum* possesses the following characteristics: unique bisaccate uterus, acraspedote strobila, testes distributed anterior to the cirrus sac, ovary C-shaped in cross-section, conspicuously enlarged genital atrium and pore (Ivanov and Campbell 2000, Jensen 2005, Jensen et al. 2016). The specimens described here as two new species of *Tetragonocephalum* from *Himantura randalli* from the Gulf of Oman are consistent with the current concept of the genus.

The genus *Tetragonocephalum* has a very controversial taxonomic history (Jensen 2005). After being erected (Shipley and Hornell 1905), between 1906 and 2000, the taxonomic status of this genus remained uncertain, accepted as valid by some authors, but only as a synonym of *Tylocephalum* Linton, 1890 by others (Ivanov and Campbell 2000, Jensen 2005). The validity of *Tetragonocephalum* was confirmed by Ivanov and Campbell (2000) in a revision of this genus and *Tylocephalum*. Further on, Jensen (2005) revised the order Lecanicephalidea, and Jensen et al. (2016) carried out a comprehensive molecular phylogenetic study of the order Lecanicephalidea. In the most recent revision of *Tetragonocephalum*, Jensen (2005) considered this genus to consist of 14 valid species.

The fourteen valid and the current two new species of *Tetragonocephalum* differ from each other based on the morphometric characteristics, including the number of testes, mature, and gravid proglottids and the size of the scolex, acetabula, testes, cirrus sac, ovary, eggs, mature and gravid proglottids.

The two new species were collected from different host individuals. One out of twenty-nine host specimens collected in May 2011 was parasitized by *Tetragonocephalum sabae* sp. n. (n = 5) and one out of seven in October 2012 by *Tetragonocephalum salarii* sp. n. (n = 4). These cestodes appear to be rare in the waters near Jod, Iran, Gulf of Oman. The occurrence of these species does not appear to be by chance, as collections were made over two years and at two different seasons. Furthermore, these host records do not appear to be aberrant or due to accidental infection because fish of the genus *Himantura* are known hosts for *Tetragonocephalum* spp. (Jensen 2005, Jensen et al. 2016), and many members of the Lecanicephalidea tend to have a high degree of host-specificity. This is often recorded for congeneric hosts; for example, *Tylocephalum* in the genus *Rhinoptera* Cuvier (see Ivanov and Campbell 2000) or *Hexacanalis* Perrenoud, 1931 in the genus *Gymnura* van Hasselt (Cielocha and Jensen 2011).

In order to compare the new and valid species, the original descriptions of valid ones were used. However, there are some limitations and uncertainties which undermine comprehensive and detailed comparisons. Some original descriptions are incomplete and lacking important morphometric data. For example, there are no appropriate measurements and/or drawings of the internal organs in the original descriptions of *Tetragonocephalum trygonis* and *Tetragonocephalum uarnak* (Shipley and Hornell 1905, 1906); Pintner (1928) introduced *Tetragonocephalum simile* as a new species without formal description and provided five drawings. Another problem in comparisons between different congeneric species of *Tetragonocephalum* is unreliable data provided in some original descriptions. Considering the descriptions of previous species, it seems that some measurements might be incorrect; for example, the maximum width of *Tetragonocephalum trygonis* (30 at scolex [probably 300?]) (Shipley and Hornell 1905). As a result, such measurements should be treated with caution. Furthermore, it appears that some features that are common to different species of the genus *Tetragonocephalum* were described differently in various species, such as absence of cephalic peduncle, spatial pattern of testes, shape of ovary, and armature of cirrus. Also, there are some features, which have not been taken into account so far and might be important in species identification, such as the attachment pattern of apical organ to scolex proper modification and strobila to scolex proper, the degrees of overlap between fields of testes and anterolateral vitelline follicles, and the histological structure of testes.

In conclusion, the problematic taxonomic status of some previously described species, due to inappropriate and/or incomplete descriptions, and type materials which are either unspecified or missing, make it necessary to designate neotypes and redescribe all previously described species from the type hosts and localities, except for *Tetragonocephalum passeyi* which is thoroughly described by Jensen (2005).

## Supplementary Material

XML Treatment for Tetragonocephalum sabae

XML Treatment for Tetragonocephalum salarii
